# FAMILY FUNCTIONING: BRIDGING THE RELATION BETWEEN GRANDFAMILY HEALTH AND WELL-BEING

**DOI:** 10.1093/geroni/igad104.0400

**Published:** 2023-12-21

**Authors:** Nancy Mendoza, Youjung Lee

## Abstract

In research, it is important to utilize theories to ask and answer questions about a specific phenomenon. In grandfamily research, there is a need to utilize theory as a bridge to understanding the relationship between family functioning and grandfamily health and well-being. In this symposium, four presentations will discuss their work related to grandfamily well-being and how it was guided by theory. In Scott and Nadorff, Bowen’s Family Systems theory is used as a framework for examining the extent to which agreement on values and beliefs moderates the relation between intergenerational emotional closeness and well-being in the grandparent generation. Findings demonstrated that affectual solidarity rating and religious ideological differences between grandparents and grandchildren influenced grandparents’ well-being. Yancura & Barnett utilized the family life course framework in their study to examine how intergenerational conflict between the grandchildren’s parents and the caregiving grandparents predicted stress and depression in grandparent caregivers. Ye and Smith incorporated the family stress model that was developed by Conger and colleagues in their study. Findings suggested parenting practice profiles related differentially to the well-being of caregivers and the emotional and behavioral outcomes of the grandchildren. Musil, Jeanblanc, Zauszniewski, and Burant used McCubbin’s Resiliency Model of Family stress to examine how demographics, family demands, problem-solving/coping, resources, and situational appraisals affect depressive symptoms in grandmother caregivers. The inclusion of theory in these studies is significant. Without theory, study findings are nothing more than a pile of findings. Theory helps organize these findings in a meaningful way. This is a Grandparents as Caregivers Interest Group Sponsored Symposium.

